# Keeping Track of *Phaeodactylum tricornutum* (Bacillariophyta) Culture Contamination by Potentiometric E-Tongue

**DOI:** 10.3390/s21124052

**Published:** 2021-06-12

**Authors:** Saverio Savio, Corrado di Natale, Roberto Paolesse, Larisa Lvova, Roberta Congestri

**Affiliations:** 1 Department of Biology, University of Rome “Tor Vergata”, 00133 Rome, Italy; saverio.savio@gmail.com; 2 PhD Program in Evolutionary Biology and Ecology, University of Rome “Tor Vergata”, 00133 Rome, Italy; 3 Department of Electronics Engineering, University of Rome “Tor Vergata”, 00133 Rome, Italy; dinatale@uniroma2.it; 4 Department of Chemical Science and Technologies, University of Rome “Tor Vergata”, 00133 Rome, Italy; roberto.paolesse@uniroma2.it

**Keywords:** microalgal cultivation contaminant monitoring, potentiometric E-tongue system, microalgal biomass control, *Phaeodactylum tricornutum*, microalgal growth phase prediction, microalgal cultures discrimination

## Abstract

The large-scale cultivation of microalgae provides a wide spectrum of marketable bioproducts, profitably used in many fields, from the preparation of functional health products and feed supplement in aquaculture and animal husbandry to biofuels and green chemistry agents. The commercially successful algal biomass production requires effective strategies to maintain the process at desired productivity and stability levels. Hence, the development of effective early warning methods to timely indicate remedial actions and to undertake countermeasures is extremely important to avoid culture collapse and consequent economic losses. With the aim to develop an early warning method of algal contamination, the potentiometric E-tongue was applied to record the variations in the culture environments, over the whole growth process, of two unialgal cultures, *Phaeodactylum tricornutum* and a microalgal contaminant, along with those of their mixed culture. The E-tongue system ability to distinguish the cultures and to predict their growth stage, through the application of multivariate data analysis, was shown. A PLS regression method applied to the E-tongue output data allowed a good prediction of culture growth time, expressed as growth days, with R^2^ values in a range from 0.913 to 0.960 and RMSEP of 1.97–2.38 days. Moreover, the SIMCA and PLS-DA techniques were useful for cultures contamination monitoring. The constructed PLS-DA model properly discriminated 67% of cultures through the analysis of their growth media, i.e., environments, thus proving the potential of the E-tongue system for a real time monitoring of contamination in microalgal intensive cultivation.

## 1. Introduction

The marine unicellular diatom *Phaeodactylum tricornutum* Bohlin is currently recognized as one of the most promising microalgae for large-scale cultivation and biotechnology exploitation. The commercial potential of this diatom derives from its ability to grow in large-scale systems while accumulating a wide spectrum of marketable bioproducts. *P. tricornutum* whole biomass can also be used profitably as feed supplement in aquaculture and animal husbandry due to its biochemical profile, approximately 36% crude proteins, 26% carbohydrates and 20% lipids under standard growth conditions [1]. In addition, high contents of the carotenoid fucoxanthin, which has beneficial effects on human health (as an antioxidant agent or food supplement) has been repeatedly demonstrated as well as its crude extract bioactivity against a range of diseases [2,3,4,5].

*P. tricornutum* is also a model diatom, probably the most studied yet with a fully sequenced genome, 27 Mb, and functional genomics largely known, this microalga can be genetically manipulated using ALE [6] CRISP/Cas9 [7]. Morphologically this species possesses a unique pleiomorphism, with four different morphotypes, oval, the most diffused in culture, fusiform, triradiate and cruciform present in natural and artificial environments, the transition of which is known to be strain-specific and mediated by environmental factors such as light, temperature, salinity and nutrient availability [8,9].

The success of *P. tricornutum* in large scale cultivation has been attributed to its robustness, adaptability and high growth rates both in indoor and outdoor culture systems, such as raceways and circular ponds, or tubular, column and flat-panel photobioreactors [10]. Besides the development and optimization of single product extractions, e.g., triacylglycerols for biodiesel or eicosatetraenoic acid, docosahexaenoic acid and fucoxanthin for nutraceuticals, ‘biorefinery’ approaches have also been implemented to valorize the single *P. tricornutum* biomass in a number of different products, with continuous downstream advancements in the alga exploitation [1,11,12,13]. This is not paralleled by the upstream process steps, where several challenges still need to be overcome in order to maintain the long-term sustainability of commercial biomass exploitation [14]. In this context, the ‘algal crop protection’ in terms of detection and control of biological pollutants in the cultivated unialgal biomass still represents a major research and industry effort, which contributes to increase the overall algal production costs [15]. Monitoring of the population dynamics of the desired alga culture in its growth system appears thus crucial because culture crashes are frequent, often due to microbial contamination via a number of different transmission routes, even in close photobioreactors. Indeed, when microalgal cultures reach the stationary phase or when their density causes a self-shading effect reducing the growth, a non-optimal condition is established, and the culture become susceptible to contamination, especially by ubiquitous, opportunistic species [16].

Up to date, several methods have been proposed to allow for increasingly rapid and accurate detection of culture contamination based on ‘microbial/biological’ composition analysis in the laboratory, in spot samples. To this end, different observational/imaging techniques (up to FlowCAM applications, [17], spectral fingerprints and also in association with artificial neural networks, ANNs, [18], and molecular diagnostics, e.g., quantitative real-time polymerase chain reaction (qPCR) [19,20] and microbiome next generation sequencing (NGS) to unveil predator or pathogen burden (NGS) [21]), were developed. In any case, these procedures focus on detecting/checking culture invasion in culture aliquots and require highly qualified staff involvement, and virtually include infrequent, discontinuous sampling and assays. Besides, a diverse perspective in algal culture management focuses on control strategies based on microbe interaction fundamentals. In fact, natural chemicals produced by marine microalgae as predator deterrents were used to control ciliate contamination in mass green alga cultures [22]. Moreover, the biological control approach was implemented to eliminate a ciliate contaminant by the addition of a carnivory, predatory copepod, with no reduction in the microalgal biomass concentration [23] and overnight CO_2_ asphyxiation was also applied to control zooplankton populations in closed and open systems [24].

Overall, there is still a need to develop effective strategies to maintain the microalgal biomass process at the desired productivity and stability, especially in terms of early warning methods able to allow early remedial actions and to undertake countermeasures (e.g., using chemical and biological, antimicrobial, agents or by modifying culture parameters in order to prevent contaminant proliferation) and to avoid culture collapse and consequent economic losses.

On our opinion, promising indications derive from the integration of the two distinct approaches pursued so far, i.e., contaminant detection versus contaminant control, providing the possibility to rapidly detect culture modifications due to unwanted ecological interactions (microbial contaminations) that result in an altered culture environment established when the population dynamics of the cultivated microalga is subject to competition, grazing or infection pressures.

With the purpose of keeping track of these ‘unknown’ and ‘aspecific’ microbial interactions in the culture media, indicative of culture contamination, the application of multisensory systems, such as Electronic tongue (or E-tongue), appears to be particularly suitable. E-tongue is an analytical instrument that employs the working principle, similar to the “chemical” senses (olfaction and taste) of mammalians. E-tongue is composed of an array of cross-sensitive sensors (not having a pronounced selectivity) to a certain group of analytes, in combination with the mathematical procedures for multivariate signal processing, such as, for instance, cluster analysis (CA), principal component analysis (PCA), regression methods and others [25,26].

Previously, E-tongue and E-nose artificial sensing systems have already been used to analyze other organisms, such as, for instance, *Aspergillus* spp. [27], dermatophytes and selected fungi [28,29] and released mycotoxins [30] among others. Recently, the E-tongue was used to monitor the fermentation process, which share with microalgae mass cultivation the small set of parameters to control (temperature, pH and CO_2_). The E-tongue measurements in combination with the PCA analysis of the recorded potentiometric signals allow us to follow the kinetics process over time [31,32,33]. Moreover, the degradation process of algal cultures was studied by means of E-tongue. In fact, the growth and after harvesting biomass degradation of *Nannochloropsis oceanica* were assessed by means of a voltammetric E-tongue and impedance spectroscopy; while the NMR spectroscopy served to register the algal chemical changes in [34], demonstrating the utility of the multisensor approach for rapid and robust quality control on microalgae production plants. Another E-tongue application is drinking water quality monitoring [35], including the detection of cyanobacterial toxins [36]. Indeed, the E-tongue approach was demonstrated to be a useful tool not only for detecting cyanobacterial toxin in potable waters, but also to distinguish in culture media between toxin-producing and non-toxic cyanobacterial strains [37].

In this work, an E-tongue system was applied to record variations in the culture environments over the growth of the diatom *Phaeodactylum tricornutum*, and of a diatom contaminant isolated from an indoor photobioreactor, as well as of their mixed culture. We demonstrate that the E-tongue can be a fast and indirect tool to monitor microalgal purity and to predict the culture growth stage, opening new perspectives for the management of intensive microalgae cultivation and prospecting implementation for real time contaminant detection. To the best of our knowledge, no other research investigating the application of artificial E-tongue sensing systems for monitoring and keeping track of possible microbial contamination over time, have yet been reported.

## 2. Materials and Methods

### 2.1. Microscopy

Morphological analysis of the cultures was performed in light (Zeiss Axioskop; Carl Zeiss Inc., Oberkochen, Germany) and confocal laser scanning microscopy (FV1000 Olympus Corp., Tokyo, Japan) at the Center of Advanced Microscopy ‘P. Albertano’, Department of Biology, University of Rome ‘Tor Vergata’. The excitation wavelength was in the red (636 nm, Ar/HeNe laser source) while frustule silica signal was recorded using the same laser source in reflection mode and was acquired using a bandpass filter (560–660 nm).

### 2.2. Microalgal Cultures and Growth Curves

The diatom strain *Phaeodactylum tricornutum* SAG 1091–1a was obtained from the Culture Collection of Algae (SAG) of the Goettingen University and maintained in Guillard’s (F/2) medium at 25 °C, 12:12 h D:L, illuminated by a white light lamp (Osram T8 EM 16.4 W 865) at the irradiance of 80 µmol photons m^–2^s^–1^. The microalgal contaminant used in this work was isolated, serial dilutions and plate streaking, from a spent culture of *Chaetoceros muelleri* (UTEX LB FD74) grown in indoor photobioreactors (8 L) as aquaculture feeding. The isolated contaminant was maintained under the aforementioned conditions.

Two unialgal cultures were prepared from the inocula of the abovementioned stock cultures at cell densities of 114 × 10^4^ cell/mL. Then, a co-culture, *Mix* co-culture, of the two strains was prepared using an inoculation ratio of 1:1, with a final cell density as above. Cultures were put in Erlenmeyer flasks with appropriate volumes of fresh F/2 medium, mixed by air insufflation. During the growth time, the three cultures were maintained at 25 °C, 12:12 h D:L and irradiance of 80 µmol photons m^–2^s^–1^. Cultures were monitored in three 1 mL sample aliquotes, collected twice a week over a 22 day period, from each culture by microscopy analysis and by recording of Optical Density (OD_680nm_ for chlorophyll *a* in vivo absorbance and OD_730 nm_ for culture turbidity) at a (ONDA UV-30 SCAN) spectrophotometer. In addition, the other three aliquots of 3 mL were sampled. One used for UV-vis spectra recording, Cary 50 spectrophotometer, while fluorescence data were acquired with a Shimadzu RF-1501 (Kyoto, Japan) fluorimeter (λ_exc_ = 470 nm). The measurements were performed in a glass cell of 1 cm path length. The other two aliquots were filtrated with 0.45 μm pore size Whatman borosilicate filters and analyzed as the other aliquots with the multisensory E-tongue system.

Two consecutive growth experiments were performed in order to monitor *P. tricornutum* and *Contaminant* cultures in unialgal conditions and in *Mix* co-culture over time. The first measurement round, used as a preliminary test to evaluate the E-tongue sensor response, was performed from 16.09.2020 to 06.10.2020 (cycle I), and the second round was run from 04.02.2021 to 02.03.2021 (cycle II).

### 2.3. Potentiometric E-Tongue System

Potentiometric sensors of four different types were included in the E-tongue system, among them: (i) sensors with PVC-based solvent polymeric membranes doped with 5,10,15,20-tetraphenyl-porphyrinato Co(III) chloride (Co(TPP)Cl, sensor A1), nonactin (sensor C1), heptyl-4-trifluoroacetylbenzoate (Carbonate Ionophore I, sensor A4) and thenoyltrifluoroacetone (TTA, sensor C6) ionophores; (ii) chalcogenide glass sensors-G4Cu, C11Pb and M6Ag; (iii) metallic sensors-Pt and Ag rods (99.99% pure), and alloys-stainless steel 304 (alloy of Fe and18% Cr, 10% Ni, 0,05% C), bronze (an alloy of Cu and 12% of Sn), Br60 (an alloy of Cu and 20 wt.% Zn), Ag + Cu alloy (Ag 60%–Cu 26%–Sn 14%), and EC60 alloy (Sn 40%–Pb 60%); (iv) polycrystalline chloride-selective sensor based on LaF_3_ (sensor A7). The PVC-based solvent polymeric membranes were prepared according to the standard method reported elsewhere [38,39]. Chalcogenide glass and polycrystalline sensors were purchased from Sensor Systems (St. Petersburg, Russia). Metallic sensors were prepared in our laboratories from metallic bars sections of 3 cm length and 3 mm in diameter, cut and incorporated in Teflon bodies, and pre-treated before and during the analysis, as described in our previous works [40,41].

The measurements with potentiometric E-tongue system were performed with LiquiLab (ECOSENS srl, Rome, Italy) high-impedance analog-to-digital potentiometer. The potentials of electrodes were measured versus a Saturated Calomel Reference Electrode (SCE, AMEL, Italy), in a standard two-electrode configuration cell. Prior to the potentiometric testing, the freshly prepared sensors with PVC-based polymeric membranes were soaked in 0.01 mol/L NaCl aqueous solution for at least 24 h and then stored in this solution between measurements. Chalcogenide glass, polycrystalline and metallic electrodes were stored in air. E-tongue array was rinsed with distilled water and carefully dried with filter paper before and after every measurement. Additionally, metallic electrodes, in order and to prevent possible contamination due to adsorption or either precipitation of poorly soluble products, were mechanically cleaned with a fine emery-paper, rinsed with ethanol and dried in air. The stability of the sensor responses was controlled through the whole experiment duration in two standard solutions: 0.01 mol/L NaCl and tap water (once sampled in sufficient amounts and used over all the experiment duration), were measured at the beginning and at the end of each experimental day. When required, the E-tongue sensors response drift correction was performed through the calculus of correlation coefficients from the sensor’s responses in standard solutions for every day of measurement. The details on E-tongue sensors drift correction according to the multivariate calibration transfer procedure can be found in [42,43]. All the measurements in microalgal cultures filtrates were repeated in triplicate. Additionally, the pH and the conductivity of the culture’s filtrates were controlled during the experiments with pH glass electrode (AMEL Instruments, model411CGG/6) and portable conductometer (HANNA, model Dist 4) respectively.

### 2.4. Data Analysis

Chemometric data analysis included identification, classification and quantitative estimation of culture variations. Identification was performed using Cluster Analysis (CA) and Principal Component Analysis (PCA) techniques. Partial Least Square Discriminant Analysis (PLS DA) and Soft independent modelling by class analogy (SIMCA) methods were applied for samples classifications. Both raw and auto scaled through the mean normalization procedure data were treated through the data analysis. Partial Least Square Regression (PLS) was used to correlate the E-tongue output to the samples fluorescence measured on the same sampling days. Data treatment was performed with a commercial Unscrambler (v9.1, 2004, CAMO PROCESS AS, Oslo, Norway). Due to the restricted number of measurements composing the dataset, a leave-one-out validation was applied. The RMSEP (Root Mean Square Error of Prediction) and correlation coefficients, R^2^, of predicted vs measured correlation lines were used to evaluate the efficiency of the constructed regression models.

## 3. Results and Discussion

### 3.1. Microscopy Observation

**Figure 1:**
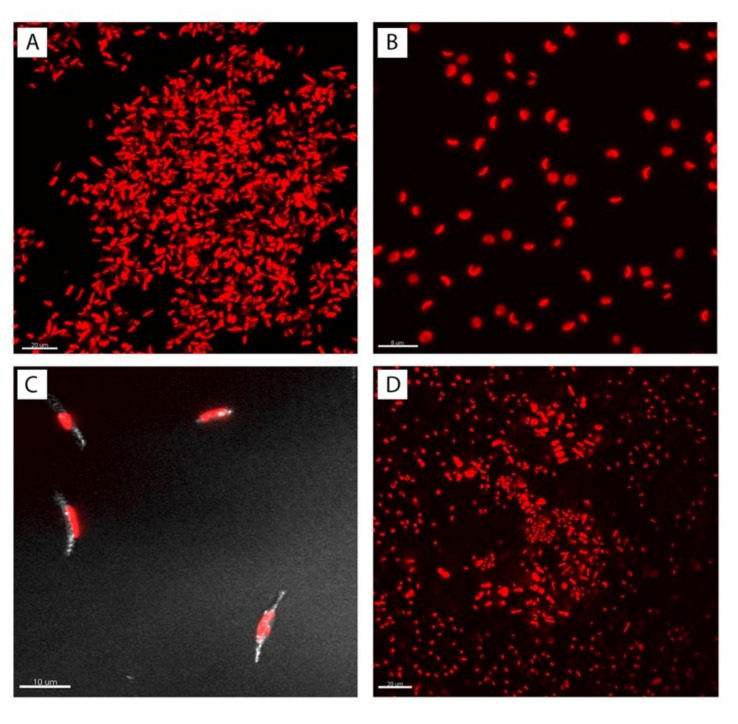
CLSM micrographs of the different cultures, red signal is due to chlorophyll *a* emission (red channel), white signal to silica cell wall autofluorescence. (**A**). *Phaeodactylum tricornutum* culture; (**B**). *Contaminant* culture; (**C**). Details of *P. tricornutum* fusiform, silica cell walls, white signal; (**D**). *Mix* co-culture of *P. tricornutum* and *Contaminant* (1:1).

Microscopy analyses were conducted to evaluate the health state of the cells in the different cultures and to circumscribe the contaminant identity. Overall, cultured cells were tiny with a rather overlapping size range. As for the cultured diatom, light microscopy observations evidenced only the *P. tricornutum* fusiform morphotype, with no morphological transition detectable in the samples collected for culture screenings. The fusiform diatom cells measured about 7–10 μ in length and were around 2.5–3 μ wide; besides, they clearly showed one orange-brown plastid, localized in the cell center. During the contaminant isolation process, small green spherical cells were observed with no flagella (coccoid habitus) and were about 2–4 μ in diameter. Confocal microscopy, CLSM, unveiled the microalgae plastid autofluorescence upon chlorophyll *a* excitation, showing that the contaminant cells possessed a single plastid, the morphology of which was cup-shaped, indicative of its attribution to marine unicellular chlorophytes (Figure 1).

### 3.2. Culture Growth

**Figure 2:**
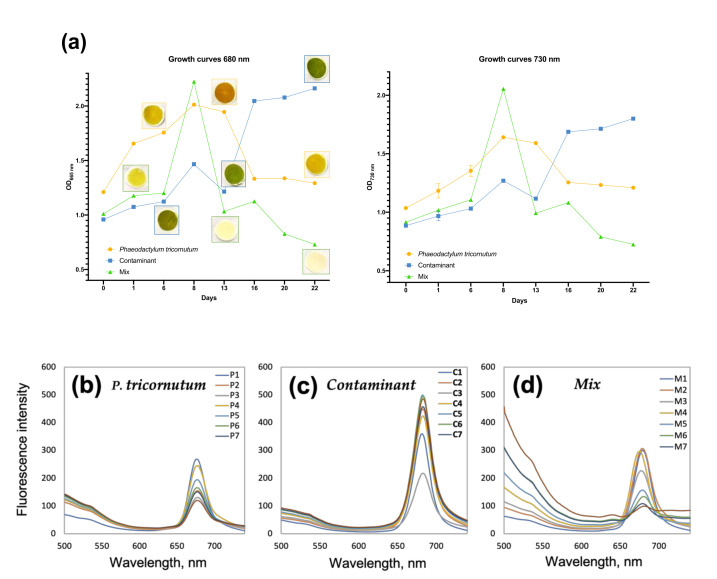
(**a**) Temporal trends of OD values at 680 and 730 nm, paper filters with filtrated cultured biomass are inserted in the 680 nm graph. (**b**–**d**) Fluorescence spectra, upon excitation at λ_ex_ = 470 nm, of (**b**) *P. tricornutum*, (**c**) *Contaminant* and (**d**) *Mix* cultures over 22 day growth.

Culture growth curves and fluorescence spectra are reported in Figure 2. As can be seen in Figure 2a, each culture has a distinctive trend. In detail, *P. tricornutum* OD values (orange line) recorded at 680 and 730 nm, increase from day 0 to day 8, indicating that growth reached its maximum at day 8. This is also clearly visible from the accumulation and color intensity, of the biomass on the filters. Then, a stationary phase followed till day 13, with no significant variations of OD values and then a decline was recorded for *P. tricornutum*. *Contaminant* culture (blue line) presents a constant increase of OD values and no plateau was observed, indicating that after 22 days of cultivation the stationary phase had not yet been reached. In contrast, the *Mix* co-culture (green line) reached the maximum of the growth at day 8 with a trend comparable to *P. tricornutum*; however, after day 8, OD values decreased sharply, indicating the collapse of the cultures as evidenced by the discoloration at day 22 of the filtrated biomass, compared to the increase in the biomass content of the pure cultures of *P. tricornutum* and *Contaminant*. This result seems to suggest that at low culture densities, the *P. tricornutum* and the contaminant can coexist and grow, while afterwards, once reached a certain density a crash in the co-culture does occur, a phenomenon also described for different biological contaminants that act as pests in algal cultures [44,45,46]. This is confirmed by analyzing the fluorescence spectra of each culture. Figure 2b–d shows the chlorophyll *a* peak (680 nm) of *P. tricornutum, Contaminant* and *Mix* cultures respectively, over the days of the experiment.

Chlorophyll *a* fluorescence intensity constant increase for the *Contaminant* in each day of sampling, indicating the constant growth of the culture over the time of the experiment. A similar trend was observed for *P. tricornutum* until day 8 (maximum day of growth), then the chlorophyll intensity decreases in according to the growth curves, which indicates that after day 8 the culture reaches the decline phase. For the *Mix*, the previously mentioned collapse of the culture, there is a clear observation of the drastic reduction of the chlorophyll *a* peak intensity starting from day 8 to day 22.

### 3.3. Monitoring Culture ‘Enviroment’ with E-Tongue

**Figure 3:**
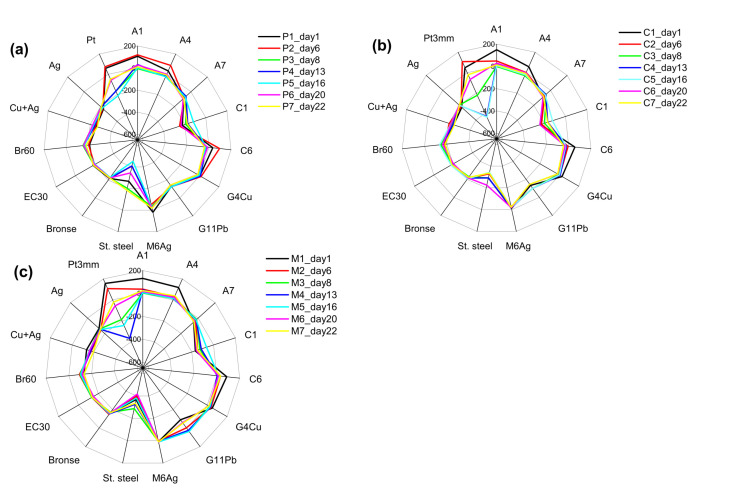
Potentiometric E-tongue data for (**a**) *P. tricornutum,* (**b**) *Contaminant,* and (**c**) *Mix* cultures filtrates over 22 day growth.

E-tongue system measurements of the three cultures are reported as radar plots in Figure 3, where the output of each single sensor is represented as an individual axis, for the two unialgal cultures and the *Mix*. Every line corresponds to the consecutive measurement day, while the potential variation range is the same for all the axes (and hence all the sensors), except for the metallic sensors, made of stainless steel and Pt, where the smaller potential range was chosen in order to better represent the differences of these sensor behaviors in different cultures. In fact, as it can be seen in Figure 3, the unialgal cultures have different profiles compared to the *Mix.*

Culture changes over growth time were reflected in E-tongue output. The most evident changes in potential over culture growth were registered for Red-Ox-sensitive metallic Pt and stainless steel sensors and for chalcogenide glass electrode G11Pb, anion-sensitive sensors A1 and A4 with organic polymeric membranes based on charged and neutral ionophores CoTPPCl and HE respectively, and especially for cationic sensors C1 (based on nonactin and sensitive to ammonia and alkali-metal ions) and C6 (based on TTA-doped polymeric organic membrane and sensitive to alkali-earth metal cations, namely to Ca^2+^ and Mg ^2+^-ions).

Based on the sensors behaviour, the significant increase in easily oxidisable species concentration, accompanied with an increase in the highly negative potentials of Red-Ox-sensitive metallic Pt and stainless steel electrodes can be deduced for all the tested samples. Besides, the differences in Pt electrode potential variation are very similar for *Contaminant* and *Mix*, while the *P. tricornutum* showed the higher variation in stainless steel electrode potential values; the same as the highest was a potential variation of cation-sensitive sensor C6 in *P. tricornutum,* Figure 3a. Compared to *P. tricornutum*, *Contaminant* culture was distinguished with the less pronounced but significantly varied potential of anion-sensitive sensors A1 and A4 and of chalcogenide-glass sensor G11Pb, Figure 3b. Moreover, this sensor evidences the influence of *Contaminant* culture on the *Mix* co-culture environment, Figure 3c. The ratio between the Red-Ox active, cationic and anionic species released in the growth media is different among different cultures.

The variation of polymeric organic membrane-based sensors A1, A4, C1 and C6 potentials may be a result of the hydrophobic algal metabolite products (that often are organic polar and non-polar molecules, such as amino acids, polypeptides, saccharides, etc.) partitioning from analyzed aqueous media and their preferable accumulation on the lipophilic sensor membrane surface; at the same time, the electrostatic interactions between metallic and chalcogenide glass electrodes and polar metabolite molecules may influence the sensor potential drop.

The obtained E-tongue profiles are well-defined and recognizable for each of the three tested cultures. Moreover, the *Mix* co-culture has shown on the one hand the characteristics common to the *P. tricornutum* and the *Contaminant*, but on the other hand that it possesses its own specificity. Nevertheless, these differences can be noticed after careful analysis of every singular sensor’s response, but unfortunately, they become inappropriate in the case when precise information on microalgal culture purity and growth dynamics should be assessed, in particular by a non-expert operator. Evidently, due to the complex biochemistry behind culture dynamics and the high number of different metabolites in a growth media, the attempt to assign the individual sensor responses to specific metabolites is not practicable and rather foolish.

**Figure 4:**
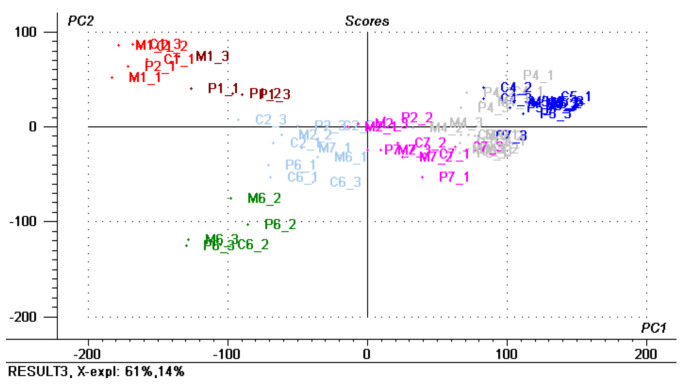
PCA scope plot of *P. tricornutum (P) Contaminant (C),* and *Mix(M)* growth over 22 days period. The colors correspond to the different clusters assigned with CA, namely: blue–class 1, red–class 2; green–class 3; light blue–class 4, brown–class 5; grey–class 6; pink–class 7.

Alternatively, the combined response of E-tongue sensors can be useful to track the culture growth and their co-contamination through chemometric modeling with user-friendly software applications, often and preferably without any qualified personnel involvement. In fact, from Figure 4, two important observations can be conducted: *i)* the different profiles of E-tongue output for the 1st day of cultures growth experiment (bold black line) indicate the potential of the multisensory system to distinguish among the *P. tricornutum* and *Contaminant* unialgal cultures and their 1:1 mixture *Mix*; *ii)* the variation of E-tongue output profiles over time follow the chemical variation of the growth media due to the various metabolites released by cultures upon proliferation and depend on the culture nature, thus suggesting the possibility of estimating the growth time (the time passed from the first day of culture growth monitoring) for mono- and co-cultures.

In order to follow and distinguish the *P. tricornutum, Contaminant*, and *Mix* co-culture according to the growth time, the cluster analysis, CA, chemometric technique was at first applied to treat the E-tongue output registered for Cycle I samples. Initially, 7 clusters, representing 7 measurement days corresponding to the 1st, 6th, 8th, 13th, 16th, 20th and 22nd day of culture growth were established; then the Euclidean distances between each of the two samples and their respective cluster centroid were estimated and the sample classes were assigned based on Sum Of Distances, SOD, values. The optimal clustering was found with the number of interactions corresponding to 50. Figure 4 shows the PCA score-plot for all tested samples with assigned clusters indicated with specific colors.

**Figure 5:**
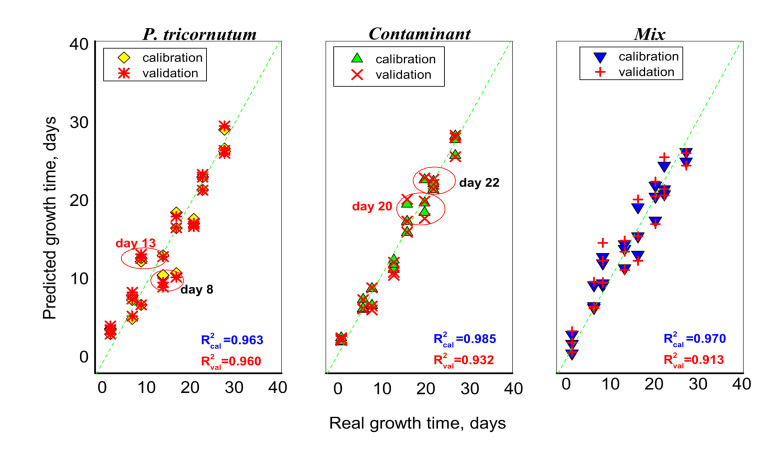
PLS correlation result for culture growth days and E-tongue response.

The measurements were repeated in triplicate in random sampling and are reported on the graph with a corresponding number of the replicates: P3_1 stays for a first replicate of *P. tricornutum* on the 3rd measurement session, corresponding to the 8th day of culture growth. As can be noticed from the Figure 4, the samples were divided into 7 classes, which correspond not only to growth time, but also distinguished among the two pure cultures and the *Mix* co-culture. Thus, the differences between the *P. tricornutum* (assigned to class 5, brown colored samples in Figure 4) the *Contaminant* and the *Mix* cultures (both assigned to the same class 2, red colored in Figure 4) was clearly evident for the first measurement day (except the misclassified sample M1_3). The difference between unialgal cultures became less evident over time as they are visible in Figure 5, while the growth temporal trend is clearly distinct especially, for all cultures, at the last week of measurements, from the fifth to seventh measurement sessions, corresponding to the 16th, 20th and 22nd days of growth (blue, green and pink colored samples respectively); while outstanding results were obtained on the 8th and 13th days of growth (grey colored samples on Figure 4). In fact, if we compare these results with those of OD and fluorescence measurements, we can confirm the observation of a significant variation in OD values and fluorescence intensities, for all tested cultures at day 8. Indeed, this day indicates a critical, turning point of culture growth as discussed above: after the sharp increase in OD values of day 8, corresponding to maximal culture growth, a clear decrease in culture densities, indicative of the culture collapse, was registered. This decline was recorded for *P. tricornutum* and *Mix* co-culture over the remaining growth time, while no significant changes and a further increase of OD (and biomass amount) were registered for the *Contaminant* (see Section 3.2 for more detailed description). The obtained results suggest, hence, the potential of non-destructive potentiometric E-tongue system to monitor and timely detect biological contamination of microalgal intensive cultures

### 3.4. Culture Growth Time Identification by E-Tongue

Culture environments can be change, over time, the cultivation, due to the interaction of target microalgal strains with the culture media, their microbioma and by other microalgal contaminant. Our hypothesis is that these interactions, which cause changes in culture environment ‘features’, also occur in *P. tricornutum* and *Contaminant* cultures, and in the *Mix* co-culture, and can be revealed by an ‘a-specifically’ by the E-tongue system, providing a set of signals useful to discriminate the cultures during the cultivation period.

A PLS regression model was built on E-tongue acquired data, in order to predict the culture growth days (represented along the X-axis) for unialgal cultures of *P. tricornutum* and *Contaminant*, and for *Mix* co-cultures during the Cycle II of the microalgae growth monitoring experiment. The model was obtained with 6 latent variables and represents 90.15% of the total system variance. As can be seen from the Figure 5, the E-tongue output data allowed a good prediction model for the culture growth time expressed as days, with R^2^ values for validation which varied in a range from 0.913 to 0.960 and RMSEV of 1.97–2.38 days.

The most robust model for E-tongue response was obtained for *Contaminant* unialgal culture, for which, anyway, some uncertainty was registered for the 20th and 22nd days of culture growth. In *P. tricornutum*, the evident data dispersion was registered for the 8th and 13th days; these days were already evidenced as critical points (day 8) of culture growth followed by the culture collapse (from day 13). The PLS model of *Mix* co-culture shows a higher dispersion and is less robust than unialgal cultures in terms of growth time estimation with R^2^ = 0.913 for validation procedure.

In order to better understand the differences in the cultures over time, the PLS regression model was integrated with the fluorescence spectra obtained from the unfiltered samples (Figure 2) and correlated with the E-tongue data in Table 1.

For *P. tricornutum* culture, E-tongue has demonstrated a good correlation to the fluorescence intensity variation at 540 and 680 nm, while for the *Contaminant,* E-tongue output signal was better correlated to the fluorescence intensities of samples, measured at 540 and 640 nm. Finally, for the *Mix* co-culture, the fluorescence intensity values at all three wavelengths were well correlated to the E-tongue output.

These results suggest that the signals detected in the filtrated culture media samples by the E-tongue present a strong correlation with wavelength values associated to the pigmentary content of microalgae, providing an important feature to discriminate microalgal cultures and contamination with a noninvasive, nondestructive and no-time consuming multisensory approach compared to the spectroscopy analysis usually used for monitoring microalgal intensive cultivation.

### 3.5. Identification of Culture Contamination by E-Tongue

**Figure 6:**
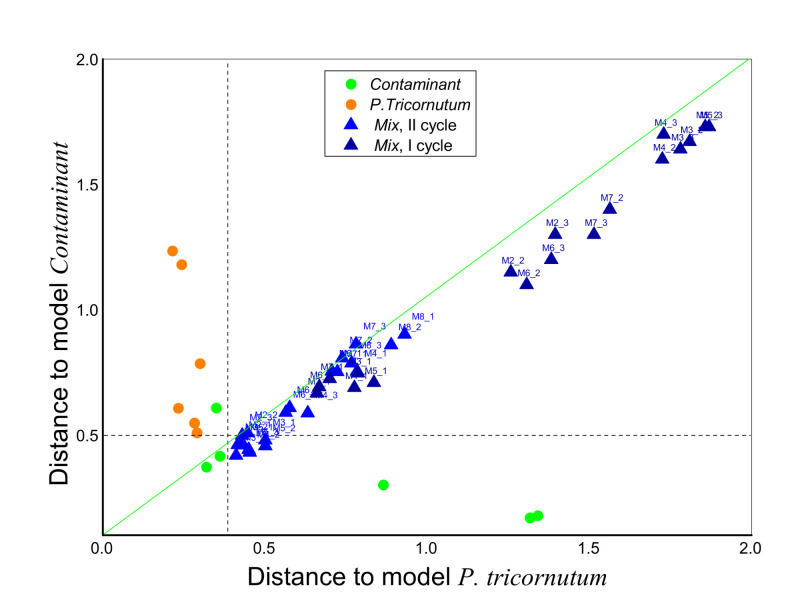
SIMCA classification Coomans plot for the *Mix* co-culture over the growth time in relation to the unialgal *P. tricornutum* and *Contaminant* cultures.

The possibility to discriminate the *Mix* co-culture from pure monocultures *P. tricornutum* and *Contaminant* was investigated with Soft independent modelling by class analogy (SIMCA), Figure 6. For this, two individual models corresponding to the *P. tricornutum* and the *Contaminant* measured for a first day of growth experiment of two measurement cycles, I and II were built first, and then the *Mix* samples at different stages of co-culture growth were classified as unknown samples. As can be seen from the Figure 6, most of the *Mix* samples were identified as not belonging to the pure cultures, and this classification was irrespective of the culture growth dynamics. Moreover, upon growth, the *Mix* co-culture resembles a higher degree to the *Contaminant*, rather than to the *P. tricornutum*, which is expressed by higher sample coordinate values along the X-axis, representing the sample distance to Model *P. tricornutum* in comparison to the shorter distances to Model *Contaminant* (scores values along Y-axis).

Furthermore, we have checked the ability of E-tongue to discriminate pure monocultures *P. tricornutum* and *Contaminant* and the *Mix* co-culture by means of PLS-DA. For the classification purposes, to each culture a separate class number was assigned. Due to the restricted dataset (36 samples measured during the measurement cycles I, II, by 15 sensors, 540 points in total) one-leave-out cross-validation of calculated PLS-DA model was employed, and the first and the last day of the culture growth experiment were considered. The obtained results are given in Table 2. Among the 36 analysed samples, two *P. tricornutum,* four *Contaminant* and six *Mix* samples (12 samples in total) were misclassified, while the other 24 samples, were correctly assigned to the corresponding class, thus resulting in discrimination of 67% of cultures through the analysis of growth media by means of the E-tongue system. This is a promising result considering the complexity of the task and the absence of the alternative fast and non-invasive in-line methods of algal biomass contamination monitoring.

Taken together, the results of the SIMCA and PLS-DA methods suggest that E-tongue is able to discriminate the Mix co-culture respect to unialgal cultures during the cultivation period, thereby prospecting E-tongue to be successfully applied for the monitoring and contaminant detection of microalgal intensive cultivation.

## 4. Conclusions

In this paper, the potentiometric E-tongue system was tested as a tool for a rapid and non-destructive estimation of variations in culture environments over the growth of *P. tricornutum* and *Contaminant* unialgal cultures and of their mixed culture. The results obtained indicate a potential utility of the E-tongue system not only for pure cultures recognition, but also to monitor microalgal culture purity and to keep the pace of a microalgal contamination, possibly through the direct introduction of the E-tongue sensors inside a photobioreactor and a continuous reading of sensor output. Such a monitoring can be easily performed inline, without any human personnel involvement, and permits a timely early warning, opening up new perspectives for the management of intensive microalgae cultivation and prospecting, promising the implementation of real time contaminant detection through the implementation of appropriate countermeasures to save the crop mass production.

## Figures and Tables

**Table 1:** PLS regression parameters between E-tongue response and fluorescence intensities of tested microalgae cultures at 540, 640 and 680 nm respectively.

Culture	PLS Parameters	Fluorescence Intensity at		
		540 nm	640 nm	680 nm
*P. tricornutum*	slope	0.816	0.770	0.907
	offset	15.1	5.2	14.96
	R^2^	0.838	0.814	0.811
	RMSEV	6.01	1.2	20.9
*Contaminant*	slope	0.954	0.888	0.489
	offset	1.54	2.59	205
	R^2^	0.948	0.880	0.333
	RMSEV	3.89	2.86	72.5
*Mix*	slope	0.913	0.929	0.921
	offset	9.19	2.48	14.55
	R^2^	0.906	0.902	0.954
	RMSEV	19.07	4.95	19.11

**Table 2:** PLS-DA confusion matrix of *P. tricornutum, Contaminant,* and *Mix* cultures classification obtained by cross validation procedure.

Expected	Predicted		
	*P. tricornutum*	*Contaminant*	*Mix*
*P. tricornutum*, 1st day	**5**	**1**	
*P. tricornutum*, 22nd day	**5**	**1**	
*Contaminant*, 1st day		**4**	**2**
*Contaminant*, 22nd day	**2**	**4**	
*Mix*, 1st day		**4**	**2**
*Mix*, 22nd day		**2**	**4**

## Data Availability

Not applicable.
